# Activation of protein kinase C in the spinal cord produces mechanical hyperalgesia by activating glutamate receptors, but does not mediate chronic muscle-induced hyperalgesia

**DOI:** 10.1186/1744-8069-2-13

**Published:** 2006-04-03

**Authors:** KA Sluka, KM Audette

**Affiliations:** 1 Graduate Program in Physical Therapy and Rehabilitation Science, Pain Research Program, Neuroscience Graduate Program, University of Iowa, Iowa City, IA 52241, USA

## Abstract

**Background:**

Protein kinase C (PKC) in the spinal cord appears to mediate chronic injury-induced pain, but not acute nociceptive pain. Muscle insult results in increased release of glutamate spinally, and hyperalgesia that is reversed by spinal blockade of NMDA and non-NMDA glutamate receptors. Therefore, we hypothesized that spinal activation of PKC 1) mediates the late phase of hyperalgesia 1 week after muscle insult, and 2) produces mechanical hyperalgesia through activation of NMDA and non-NMDA glutamate receptors.

**Results:**

Rats were implanted with intrathecal catheters for delivery of drugs directly to the spinal cord. Mechanical withdrawal thresholds of the paw were determined using von Frey filaments. Intrathecal phorbol 12,13 dibutyrate (PDBu) produced a dose-dependent decrease in the mechanical withdrawal threshold of the paw that was prevented by pretreatment with the PKC inhibitor, GF109203X. Pretreatment with an NMDA receptor antagonist (AP5) or a AMPA/kainate receptor antagonist (NBQX) prevented the decrease in mechanical withdrawal threshold by PDBu. Two injections of acidic saline in the gastrocnemius muscle decreased the mechanical withdrawal thresholds of the paw bilaterally 24 h and 1 week after the second injection. However, blockade PKC in the spinal cord had no effect on the decreased withdrawal thresholds of the paw when compared to vehicle controls.

**Conclusion:**

Spinal activation of PKC produces mechanical hyperalgesia of the paw that depends on activation of NMDA and non-NMDA receptors. Chronic muscle-induced mechanical hyperalgesia, on the other hand, does not utilize spinal PKC.

## Background

Protein kinase C activation involves translocation from the cytosol to binding domains at cell membranes of dorsal horn neurons of the spinal cord [1,2]. There are at least twelve isoforms of PKC. Several of these isoforms are concentrated in the superficial laminae of the dorsal horn, an anatomical indication that these PKC isoforms play a potential role in nociceptive signaling. In particular, PKCβ I, PKCβ II, and PKCα are found in cell bodies within the superficial dorsal horn, where PKCγ is primarily found in cell bodies in lamina IIii [2,3]. PKC is involved in many aspects of cellular sensitization, including modulation of channel conductivity by phosphorylation, increased trafficking of receptors to the cell membrane, and release of excitatory neurotransmitters [4-9].

Activation of PKC with phorbol esters in the spinal cord decreases heat and mechanical withdrawal thresholds, increases glutamate release in the spinal cord, and sensitizes spinothalamic tract and other dorsal horn neurons [9-12]. PKC is involved in animal models of both neuropathic and inflammatory pain. In rats with neuropathic pain produced by spinal nerve ligation or sciatic nerve ligation the mechanical hyperalgesia is reversed by intrathecally administered PKC inhibitors and reduced in PKCγ knockout mice when compared to wild-type mice [3,13-15]. Similarly, spinal blockade of PKC reverses the hyperalgesia induced by subcutaneous formalin, pancreatitis, thermal injury, cutaneous capsaicin, diabetic neuropathy and subcutaneous bee venom [10,16-20]. In PKCγ knockout mice acute responses to thermal and mechanical stimuli are similar to wild-type mice [3], suggesting a role for PKC in more chronic injury-induced pain, but not in acute nociceptive pain.

Activation of cAMP (cyclic adenosine monophosphate) spinally activates intracellular pathways that results in sensitization of spinal neurons and mechanical hyperalgesia. A decrease in mechanical paw withdrawal threshold produced by intramuscular injections of acid or capsaicin is reversed by spinal blockade of cAMP-PKA pathway in the spinal cord 24 h, but not 1 week, following muscle insult [21,22]. These studies demonstrate a role of the cAMP-PKA pathway in the early phase of development, but not in the later phase. We further show an increased release of glutamate in the spinal dorsal horn, and that blockade of NMDA and non-NMDA glutamate receptors 1 week after muscle insult [23,24]. Since PKC appears to mediate more chronic pain conditions we hypothesized that activation of PKC mediates the late phase of hyperalgesia 1 week after muscle insult. We further hypothesized that activation of PKC in the spinal cord produces mechanical hyperalgesia through activation of NMDA and non-NMDA glutamate receptors.

## Results

**Figure 1:**
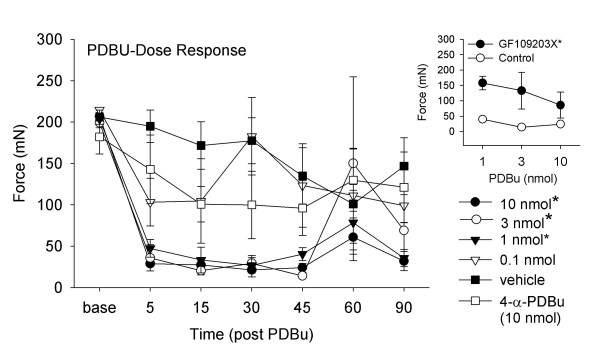
Time course of effects of intrathecal treatment with PDBu, 0.1–10 nmol/10 ml, compared to vehicle and the inactive compound 4-α-PDBu. The withdrawal thresholds for 1, 3 and 10 nmol PDBu groups are significantly less than vehicle controls or 4-a-PDBu. Inset shows that GF109203X. 0.5 nmol/10 ml, significantly prevents the decreases in withdrawal thresholds that normally occurs after intrathecal delivery of 1–3 nmol PDBu. Data are represented as mean +/- S.E.M. *, significantly different from vehicle controls.

Intrathecal injection of PDBu decreased the mechanical withdrawal threshold bilaterally in a dose-dependent manner (Fig. 1). PDBu produces a significant decrease in paw withdrawal threshold for 60–90 minutes after intrathecal injection (F_4,13 _= 9.8, p = 0.001, P = 0.001). A significant decrease was observed for doses ranging from 1–10 nmol/10 μl after injection of PDBu when compared to vehicle controls (1 nmol p = 0.004; 3 nmol p = 0.02; 10 nmol p = 0.002)(Fig. 1).

Pretreatment with the PKC inhibitor (0.5 nmol/10 μl) GF109023X prevented the decrease in mechanical withdrawal threshold produced 30 min after intrathecal injection of PDBu (1–10 nmol/10 μl)(p = 0.009) when compared to PDBu alone (Fig. 1). Pretreatment with 60 nmol/10 μl NPC15437 or 25 nmol/10 μl chelerythrine chloride had no effect on the 10 nmol/10 μl PDBu-induced decrease in mechanical withdrawal threshold. Mechanical withdrawal threshold for NPC15437 + PDBu were: 67 mN 15 min; 77 mN 30 min; 49 mN 45 min; 31 mN 60 min. Mechanical withdrawal threshold for chelerythrine chloride + PDBu were: 20 mN 15 min; 57 mN 30 min; 14 mN 45 min; 27 mN 60 min. The mechanical withdrawal thresholds after intrathecal injections of 4α-PDBu (1–10 nmol/10 μl) were significantly greater than those following PDBu injections (1–10 nmol/10 μl, p = 0.0001). Figure 1 shows the time course of effect for 10 nmol 4α-PDBu. Withdrawal threshold for the 3 nmol dose of 4α-PDBu were: 104 mN 15 min; 106 mN 30 min; 112 mN 45 min; 120 mN 60 min. Withdrawal threshold for the 1 nmol dose of 4α-PDBu were: 137 mN 15 min; 148 mN 30 min; 143 mN 45 min; 126 mN 60 min.

A significant difference between groups occurred for the changes in mechanical withdrawal threshold for the animals pretreated intrathecally with the glutamate receptor antagonists or vehicle for the 3 nmol dose of PDBu (χ^2 ^= 10.4, p = 0.005) and the 10 nmol dose of PDBu (χ^2 ^= 7.4, p = 0.02). Specifically, pretreatment with AP5 prevented the decrease in mechanical withdrawal threshold produced by PDBu when compared to animals receiving vehicle (3 nmol/10 μl n = 5, p = 0.006; 10 nmol/10 μl; n = 5, p = 0.006). Similarly, pretreatment with the non-NMDA receptor antagonist NBQX prevented the decrease in mechanical withdrawal threshold following PDBu when compared to vehicle controls (3 nmol/10 μl, n = 5, p = 0.006; 10 nmol/10 μl, n = 8, p = 0.03). There was a smaller effect of NBQX on the hyperalgesia produced by 10 nmol PDBu when compared to that produced by the 3 nmol dose (F_1,12 _= 4.6, p = 0.053).

Two intramuscular injections of pH 4 saline 5 days apart produced a bilateral decrease in mechanical withdrawal thresholds 24 h and 1 week after the second injection. Spinal blockade of PKC with GF109023X (0.05–0.5 nmol/10 μl) had no effect on the decreased mechanical withdrawal threshold when delivered intrathecally 24 h or 1 week after the second injection of acidic saline compared to vehicle controls (Fig. 3).

## Discussion

The current study demonstrates that activation of PKC in the spinal cord produces a decrease in mechanical withdrawal threshold that is prevented by blockade of PKC with GF109023X. This PKC-mediated decrease in mechanical withdrawal threshold is consistent with previous data showing a similar decrease in mechanical withdrawal threshold by spinal activation of PKC [9,10]. We further show that the decreased mechanical withdrawal threshold produced by spinal activation of PKC is prevented by spinal blockade of NMDA receptors and AMPA/kainate receptors. There are several possibilities that could explain how PKC activation produces its effects through glutamate receptors. First, PKC could produce increased release of glutamate into the spinal cord resulting in continued activation of glutamate receptors. Spinal activation of PKC increases release of glutamate in vivo [9] and in vitro [12], and formalin-induced release of glutamate is prevented by blockade of PKC [17]. Further, spinal activation of PKC enhances responses of dorsal horn neurons to NMDA and AMPA agonists [12,25,26]. Thus increased glutamate release and increased activation of glutamate receptors are likely results of activation of PKC.

Second, PKC could phosphorylate the NMDA or AMPA/kainite receptor to result in increase channel conductance or potentiation of glutamate gated currents that would be manifested as an increased excitability of the neuron [27-30]. Activation of PKC sensitizes dorsal horn neurons exhibited as an increased spontaneous firing, increased response to peripherally applied mechanical stimuli [31], and an increased response to glutamate and NMDA [12]. Further, PKC decreases Mg2+ affinity in the NMDA receptor pore which increases the probability of the channel opening [26]. Indeed, in animal models of pain there is an increase in phosphorylation of the NR1 subunit and GlurR1 subunit that likely depends on activation of PKC [32-36].

Third, phosphorylation of NMDA or AMPA receptor subunits could increase trafficking and insertion of receptors into the cell membrane and synapse. Phosphorylation of NR1 by PKC increases surface expression of NMDA receptors, increases delivery of NMDA receptors to the surface and to the synapse [28,37,38]. The AMPA receptor subunits are also phosphorylated by PKC [27,29]. However, the functional role of phosphorylation of AMPA receptor subunits in receptor trafficking has not been tested to date. Thus, increased phosphorylation of glutamate receptors could result in an increased channel conductance and an increased number of receptors available synaptically resulting in increased excitation of the nociceptive spinal neurons.

Lastly, PKC decreases efficacy of inhibitory neurotransmitters on spinothalamic tract neurons, which would be manifested as an increased excitation. For example intradermal injection of capsaicin reduces the inhibition of spinothalamic tract neurons normally produced by electrical stimulation of the periaqueductal gray or by GABA; this loss of inhibition is prevented by spinal blockade of PKC [39]. Similarly, the inhibition of calcium channels by μ-opioid agonists in the spinal dorsal horn of rats is prevented by inhibition of PKC [40]. Thus, increased PKC activity reduces normal inhibition within the spinal cord. Thus, spinal activation of PKC could result in increased glutamate release, increased channel conductance of glutamate receptors, increased number of glutamate receptors in the membrane, and decreased inhibition. Taken together this would result in increased excitability of neurons that is manifested as decreased withdrawal thresholds to noxious stimuli.

Surprisingly, spinal blockade of PKC had no effect on the decreased mechanical withdrawal threshold induced by repeated intramuscular acid injections suggesting PKC activation in the spinal cord does not maintain the hyperalgesia once developed. However, the current study did not determine the role of PKC activation in the development of the hyperalgesia and future studies should investigate if pre-treatment with PKC inhibitors prevents development of hyperalgesia. The lack of effect of PKC on the maintenance, however, is inconsistent with previous studies that show nocifensive behaviors and dorsal horn neuron sensitization are reversed by PKC inhibitors in models of inflammatory and neuropathic pain [3,10,11,13-20,41]. Mice lacking PKCγ show a significant deficit in the hyperalgesia associated with neuropathic pain and the second phase of the formalin test [3]. Further, the number of neurons staining for PKCγ increases in the spinal cord in animals with neuropathic pain [42], PKCγ staining increases in the spinal cord following CFA-paw inflammation that lasts through 14 days [43], PKCβ II is increased in the cell membranes in the spinal cord following CFA-inflammation through 14 days [2], and phosphorylated PKC increased in the spinal cord after thermal injury [44]. Taken together, it appears that PKC is involved in long-term hyperalgesia, and that PKCγ and PKCβ II may be important in the processing of nociceptive information in chronic hyperalgesia. However, blockade of PKC in the spinal cord had no effect on the acid-induced model of muscle pain. This model is unique; there is no detectable injury to the peripheral muscle after injection of acidic saline and hyperalgesia is maintained by changes in the central nervous system [45]. Thus, differences could be related to the dependence on continued primary afferent input to the spinal cord. Differences could also result from the type of tissue activated: i.e. cutaneous vs. deep somatic tissue. Prior studies utilize animal models with cutaneous inflammation, i.e. formalin, capsaicin, paw carrageenan, paw CFA, cutaneous thermal injury, or nerve injury which likely includes cutaneous afferent damage. The mechanisms responsible for cutaneous pain are distinctly different from those related to muscle pain. In support, we previously show that capsaicin injected into cutaneous tissue results in short lasting (hours) mechanical hyperalgesia while capsaicin injected into muscle or joint results in long-lasting mechanical hyperaglesia (days to weeks) [21]. Formalin injected into skin of the back resulted in fos expression in laminae I-V; whereas formalin injected into the paraspinal muscles did not result in fos staining in laminae II [46]. Formalin injection into the muscle, when compared to the skin, resulted in greater fos expression in brain areas known to mediate descending facilitation of nociception, i.e. amygdala, and the ventrolateral periaqueductal gray II [46]. Thus different anatomical pathways could mediate differences between muscle pain and those in other cutaneous pain models.

The current study showed that 2 or the 3 PKC inhibitors tested were ineffective against PDBu, an activator of PKC. GF109023X has been shown to be more efficacious in inhibition if substance P-induced nocifensive behaviours compared to chelerythrine chloride [47]. In the current study, GF109023X significantly reduced the effects of 10 nmol PDBu; while chelerythrine chloride and NPC15437 had no effect on the reduction in withdrawal thresholds produced by NPC15437. It is possible, based on the differences in efficacy of the PKC inhibitors, that an effect could be observed if tested against lower doses of PDBu. Of note, the majority of the previous studies did not test the PKC inhibitor utilized against the agonist. The doses of PKC inhibitors utilized in the current study, were similar or higher than those utilized previously [47-50]. However, dosing with chelerythrine chloride and NPC15437 were limited by solubility for in vivo delivery of the antagonist to the spinal cord intrathecally. Our prior study using microdialysis was able prevent the effects of activation of PKC with NPC15437 [10]. This different methodology may be able to deliver a higher effective dose closer to the neurons than a single intrathecal bolus since it utilizes continued slow diffusion into the spinal cord parenchyma.

Although the current data support that blockade of glutamate receptors prevents the onset of PKC-induced hyperalgesia, it is possible that these glutamate receptor antagonists were analgesic on their own and thus masking the effects of PDBu. One limitation to testing mechanical thresholds with von Frey filaments is that we cannot detect an analgesic effect as the cut-off for withdrawal is essentially our baseline response before injury. The doses of AP5 and NBQX used in the current study, however, have no significant effects on motor behaviors 15–120 minutes after delivery of the drug intrathecally [23,51,52]. Further, intrathecal treatment using AP5 (10 nmol), similar doses to those in used in the current study, had no effect on the hyperalgesia that develops after plantar incision [52,53]. These data argue against the analgesic potential of these antagonists at the doses utilized, and thus support our conclusions that PDBu produces its effects through activating NMDA and non-NMDA receptor antagonists.

**Figure 2:**
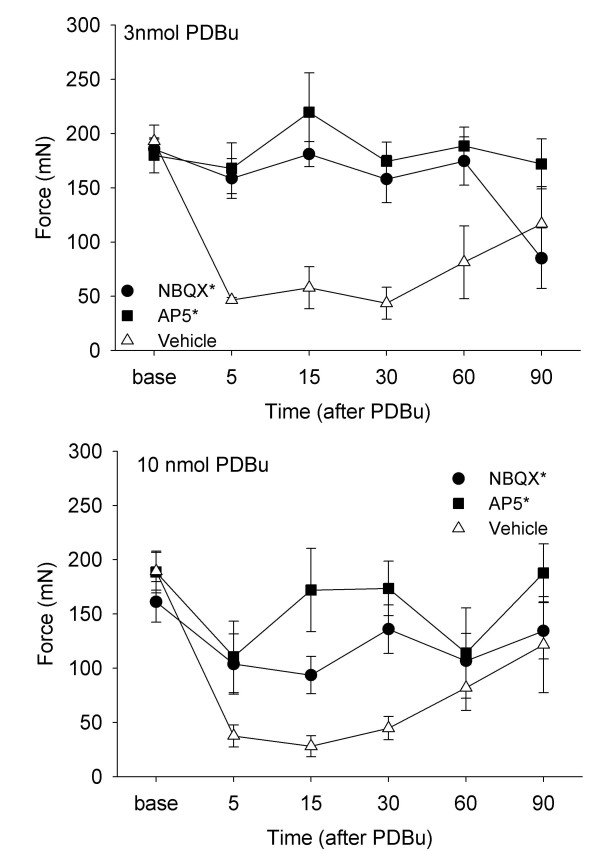
Time course of effects of intrathecal pretreatment with AP5 or NBQX on the decreased withdrawal threshold induced by PDBu, for 3 nmol dose (top graph) and for the 10 nmol dose (bottom graph). Data are represented as the mean +/- S.E.M. *, significantly greater than vehicle control

**Figure 3:**
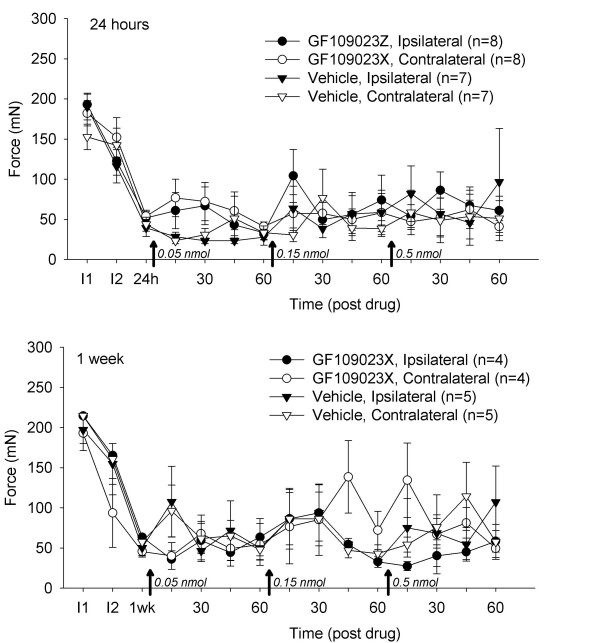
Effects of GF109203X, compared to vehicle, on the decreased withdrawal thresholds 24 h and 1 week after repeated intramuscular acid injections. Data are the mean +/- S.E.M.

## Conclusion

Activation of the PKC pathway in the spinal cord produces mechanical hyperalgesia that is prevented by blockade of PKC, NMDA, or AMPA/kainite receptors. However, activation of PKC in a model of non-inflammatory muscle pain has no role in the maintenance of the hyperalgesia.

## Methods

All experiments were approved by the animal care and use committee at the University of Iowa and are in accordance with NIH guidelines and the International Association for the Study of pain. A total of 123 male Sprague-Dawley rats (250–450 g; Harlan, St. Louis, MO) were utilized in these studies.

### Intrathecal catheter placement

Animals were placed under general anesthesia using 2% halothane. Catheters (32 G polyeurethane tubing, 10 cm; ReCathCo, Allison Park, PA) were attached to PE10 tubing (0.28 mm ID, polyeurethane, 10 cm; Becton Dickson and Company, Sparks, MD) and threaded through a 23 G needle. The 23 G needle was inserted into the intrathecal space between L5/L6 until a tail flick was elicited confirming placement intrathecally. The tubing was then advanced 4 cm so the tip was located in the lumbar enlargement. The catheter was secured to the fascia, the PE10 tubing was threaded out between the shoulder blades, and the incision was closed with silk sutures. Animals were allowed to recover from anesthesia under observation prior to testing 3–5 days later.

### Mechanical withdrawal threshold

Before behavioral testing, rats were placed in clear Plexiglas™ chambers on a wire mesh elevated surface to acclimate for 30–40 min. Withdrawal thresholds from mechanical stimuli of von Frey filaments of ascending bending force (from 10.0 to 494.6 mN) were applied twice to the plantar surface of the bilateral hind paws. A positive response was defined as withdrawal from the von Frey filament. Confirmation of threshold was tested by examining the filament above and below the withdrawal response. Test-retest reliability was previously determined for this method (*r*^2 ^= 0.7; *p *= 0.007) [54]. A decrease in mechanical withdrawal threshold is interpreted as mechanical hyperalgesia.

### Drug administration

Drugs were administered to awake animals through intrathecal (i.t.) catheters. A 30 G drug delivery needle attached to a length of PE50 tubing was affixed to the end of a 50 μl Hamilton syringe for drug administration through i.t. catheters. Drugs were delivered as a 10 μl bolus. Following the completion of all experiments correct catheter placement was confirmed by intrathecal injection of 10 μl of 2% lidocaine followed by 10 μl methylene blue dye. A catheter was considered correctly placed if there was loss of sensory and motor function after lidocaine and methylene blue dye staining included the L4-L6 spinal segments.

### Drugs

During the course of these experiments the following drugs were used: phorbol 12,13 dibutyrate (PDBu; Calbiochem, LaJolla, CA; 0.1 nmol/10 μl, 1 nmol/10 μl, 3 nmol/10 μl, 10 nmol/10 μl), a phorbol ester that directly activates the PKC pathway by mimicking diacylglycerase; 4α PDBu (Calbiochem, La Jolla, CA; 1 nmol/10 μl, 3 nmol/10 μl, 10 nmol/10 μl), the inactive isoform of PDBu; GF109203X (Sigma Chemical Co., St. Louis, MO; 0.5 nmol/10 μl, 0.15 nmol/10 μl, 0.05 nmol/10 μl), a non-selective PKC inhibitor [14,47]; chelytherine chloride (Sigma Chemical Co., St. Louis, MO; 25 nmol/10 μl), a non-selective PKC inhibitor; NPC15437 (60 nmol/10 μl) a non-selective PKC inhibitor; AP5 (Sigma Chemical Co., St. Louis, MO; 20 nmol/10 μl), an N-methyl-D-aspartate (NMDA) receptor antagonist; NBQX (Sigma Chemical Co., St. Louis, MO; 10 nmol/10 μl), an alpha-amino-3-hydroxy-5-methyl-4-isoxazole propionate (AMPA)/kainate receptor antagonist [23].

### Induction of muscle-induced mechanical hyperalgesia

Two single 0.1 ml injections of saline (pH = 4), 5 days apart, were injected into the left gastrocnemius muscle of lightly anesthetized rats (2% halothane) [45].

### Experimental Protocol

#### Experiment 1

Phorbol 12,13 dibutyrate (PDBu) (0.1 nmol/10 μl, n = 5; 0.3 nmol/10 μl, n = 4; 1.0 nmol/10 μl, n = 4; 3.0 nmol/10 μl, n = 5; 10.0 nmol/10 μl, n = 5) was injected intrathecally into the spinal cord. Paw withdrawal threshold was measured before and 30 min after intrathecal injection of PDBu. An equivalent volume of vehicle (saline with 10% DMSO) was injected instead of PDBu as a control (n = 5). As an additional control, rats were injected the inactive compound, 4α-PDBu (1 nmol/10 μl, n = 4; 3 nmol/10 μl, n = 4; 10 nmol/10 μl, n = 4).

#### Experiment 2

The ability of the PKC inhibitors GF109023X (0.5 nmol/10 μl), NPC15437 (60 nmol/10 μl, n = 4), and chelerythrine chloride (25 nmol/10 μl, n = 2) to block the decreased mechanical withdrawal threshold produced by PDBu (10.0 nmol/10 μl, n = 5; 3.0 nmol/10 μl, n = 5; 1.0 nmol/10 μl, n = 5) was tested. Ten minutes prior to PDBu administration a single dose of PKC inhibitor was given. Mechanical withdrawal threshold was tested before, 30 and 45 min after PDBu injection.

#### Experiment 3

The ability of AP5, an NMDA receptor antagonist, and of NBQX, an AMPA/kainate receptor antagonist, to block the decreased mechanical withdrawal threshold of the phorbol ester PDBu (3 nmol/10 μl and 10 nmol/10 μl) was tested. Initial experiments tested the effects of AP5 and NBQX on the 10 nmol/10 μl. Because of the increased variability with NBQX with the highest dose of PDBu, we tested a lower dose of 3 nmol PDBu. After baseline mechanical thresholds were determined, AP5 or NBQX were administered intrathecally 10–15 min prior to intrathecal PDBu. Rats were randomly assigned to the following groups: 1) 20 nmol/10 μl AP5 pretreatment to intrathecal 10 nmol/10 μl PDBu (n = 5), 2) 10 nmol/10 μl NBQX pretreatment to intrathecal 10 nmol/10 μl PDBu (n = 8), 3) vehicle pretreatment (saline) to intrathecal 10 nmol/10 μl PDBu (n = 5), 4) 20 nmol/10 μl AP5 pretreatment to intrathecal 3 nmol/10 μl PDBu (n = 5), 5) 10 nmol/10 μl NBQX pretreatment to intrathecal 10 nmol/10 μl PDBu (n = 5), and 6) vehicle pretreatment (saline) to intrathecal 10 nmol/10 μl PDBu (n = 5). Paw withdrawal from von Frey filament stimuli was recorded bilaterally before injection and 30 min after intrathecal phorbol ester injection.

#### Experiment 4

The ability of the PKC inhibitor GF109023X (0.5 nmol/10 μl, 0.15 nmol/10 μl/10 μl, 0.05 nmol/10 μl) to block the bilateral secondary mechanical hyperalgesia produced by repeated intramuscular injections of pH 4 saline was tested in two separate groups either 24 h or 1 week after the second intramuscular injection of acidic saline. Intrathecal catheters were implanted 3–5 days prior to the first injection of acidic saline and vehicle or GF109023X was delivered cumulatively to the spinal cord dorsal horn in 1 h intervals. Withdrawal thresholds were assessed before injection 1, before injection 2, 24 h after intramuscular injection of acidic saline, and after intrathecal injection of vehicle (saline, n = 5) or GF109023X (0.05 nmol/10 μl, 0.15 nmol/10 μl, 0.5 nmol/10 μl (n = 8). Withdrawal thresholds were assessed before injection 1, before injection 2, 1 week after intramuscular injection of acidic saline and after intrathecal injection of saline (n = 5), GF109023X (0.05 nmol/10 μl, 0.15 nmol/10 μl, 0.5 nmol/10 μl (n = 4). The tester was blinded to treatment group. Mechanical withdrawal threshold was assessed 15, 30, 45, and 60 min after the injection of drug or vehicle.

### Data analysis

Paw withdrawal thresholds were compared across time, and between groups, using statistical analysis with a repeated measures analysis of variance (ANOVA). Post hoc testing between individual groups was done using a Tukey's test. Significance was determined at p ≤ 0.05. Data is presented as the mean +/- S.E.M.

## Competing interests

The author(s) declare that they have no competing interests.

## Authors' contributions

KMA carried out all the studies outlined in the manuscript and participated in the design of the studies. KAS conceived of the study, participated in the design and coordination of the studies, performed statistical analysis, and wrote the initial draft of the manuscript. Both authors read and approved the final manuscript.
